# Cholesterol and Haemolysis

**Published:** 1915-08

**Authors:** 


**Cholesterol and Haemolysis.** Jacob Rosenbloom and James P. McKelvy of Pittsburg, Interstate Med. Jour., February, 1915, in reference to a case of haemolytic jaundice with splenomegaly, state that if erythrocytes are carefully washed to free them from serum, cobra venom, tetanus toxin, solanin, saponin, etc. fail to cause solution of the corpuscles. One of the most characteristic findings in haemolytic jaundice is the lessened resistance to hypotonic sodium chlorid solution. Chauffard has found that normally, haemolysis begins with 0.44 per cent. salt solution and is complete with 0.36 per cent In the present case, haemolysis was very marked with solutions even as strong as 0.6 per cent., was present up to 0.65 per cent., but failed to occur at 0.9 per cent. The patient was put on a diet consisting of approximately 60 grams protein, 75 fat, 112 carbohydrate, totalling 1395 calories. For a period of 5 days, the cholestrol intake was 5.22, the excretion in the faeces 12.27 grams, an excess of 7.05 grams. It has been known that lecithin accelerates and that cholestrol inhibits haemolysis. Hence it is suggested that the characteristic pathogeny of haeloytic jaundice depends upon a lessening of the latter, protective agent, in the system. Medak, Biochem, Zeitschr. page 419, vol. 59, 1914, gives the following table:

| Disease | Free cholesterol in 1,000 c.cm. blood grm. | Combined cholesterol in 1,000 c.cm. blood grm | Total cholesterol in 1,000 c.cm. blood grm |
|---|---|---|---|
| Catarrhal jaundice .............. | 0.8377 | 0.4150 | 1.2572 |
| Icterus gravis (mechanical)....... | 1.3900 | 0.4917 | 1.8817 |
| Jaundice (on admission) ......... | 1.8200 | 0.8856 | 2.7056 |
| Jaundice (after disappearance of jaundice) .................. | 1.2600 | 0.7190 | 1.9790 |
| Pernicious anemia ............... | 0.7537 | 0.6560 | 1.4097 |
| Anemia (tuberculosis) ........... | 0.6887 | 0.3234 | 1.0121 |
| Polycythemia (cardiac) .......... | 1.0065 | 0.3310 | 1.3375 |
| Polycythemia .................... | 1.0960 | 0.4640 | 1.5600 |
| Hypertrophic cirrhosis ........... | 0.4855 | 0.6360 | 1.1215 |
| Hypertrophic cirrhosis ........... | 2.6040 | 1.0850 | 3.6890 |
| Banti's disease before splenectomy | 0.9736 | 0.3480 | 1.3216 |
| Banti's disease after splenectomy.. | 1.3980 | 0.7356 | 2.1336 |
| Banti's disease before splenectomy | 0.9510 | 0.5470 | 1.4980 |
| Banti's disease after splenectomy.. | 1.0720 | 0.7140 | 1.7860 |
| Hemolytic icterus before splenectomy | 0.4910 | 0.6330 | 1.1240 |
| Hemolytic icterus after splenectomy | 0.9560 | 0.3460 | 1.3020 |

